# 

**Published:** 1902-05-03

**Authors:** 


### NOTICE TO CORRESPONDENTS.

All MSS., Letters, Books for Review, and other matters intended for the Editor should be addressed The Editor, "The Hospital," The Hospital Building, 28 & 29 Southampton Street, Strand, W.C.

The Editor cannot undertake to return rejected MS., even when accompanied by stamped directed envelope.

**Notice to Advertisers and Subscribers.**

All Advertisements, Orders for Copies of The Hospital, and Business Communications should be addressed to **THE MANAGER, (Not to the Editor)**, The Hospital, 28 & 29 Southampton Street, Strand, London, W.C.

The Cost of Subscription, post free, is as follows.—Per week, 2½d.; per quarter, 2s. 8d.; per half-year, 5s. 3d.; per annum, 10s. 6d. Foreign:—Per annum, 15s. 2d., payable, in all cases, in advance.

**NOTICE.—Vol. XXXI. of "The Hospital" (October 1901, to March 1902), in handsome cloth binding will shortly be on sale at the office of this Journal, price, 7s. 6d. Cases for binding the Numbers contained in this Volume 1s. 6d. Index to Vol. XXXI., 2½d., post free.**

**The Monthly Part for May is now ready. Price 6d., by post 8d.**

### The Student of Medicine.

### APPOINTMENTS.

**H. Haward Bywater**, M.B.Vict., appointed House Surgeon to the Central London Ophthalmic Hospital. **Albert Carless**, M.B., M.S.Lond., F.R.C.S., Consulting Surgeon to the St. John's Hospital, Twickenham, and Honorary Consulting Operative Surgeon to the Ealing Cottage Hospital. **E. Emrys-Roberts**, M.B., Ch.B.Vict., appointed Assistant Medical Officer to Mill Road Infirmary, Liverpool. **George Geddes**, M.B., C.M.Aberd., appointed Medical Officer to the Heywood Post Office. **Alexander Harper**, M.D.Durh., M.R.C.S., appointed Assistant Medical Officer to the Princess Alice Memorial Hospital, Eastbourne. **James Holmes**, M.D.Edin., appointed Certifying Factory Surgeon for Heywood. **A. W. Lemarchand**, M.R.C.S., L.R.C.P.Lond., appointed Honorary Medical Officer to the North Devon Infirmary, Barnstaple. **R. R. W. Logan**, M.R.C.S.Eng., L.S.A., appointed Certifying Factory Surgeon for the Ashby-de-la-Zouch District of Leicestershire. **M. V. McKechnie**, L.R.C.P., L.R.C.S.Edin., appointed District Medical Officer of the Forden Union. **Lionel L. Preston**, M.B., B.S.Durh., appointed Assistant Surgeon to the Royal Isle of Wight County Hospital. **Alonzo G. Rider**, M.B.Lond., appointed Surgeon to the Royal Albert Hospital, Devonport. **W. J. H. Sinclair**, M.B., C.M.Aberd., appointed Medical Officer to the Barlinnie Prison. **Reginald Smith**, M.R.C.S., L.R.C.P.Lond., appointed Honorary Assistant Surgeon to the Warrington Infirmary. **Florence E. Willey**, M.B., B.S., B.Sc.Lond., appointed Pathologist to the Royal Free Hospital, Gray's Inn Road.

